# Address at the Inauguration of the New York State Veterinary College

**Published:** 1896-11

**Authors:** James Law

**Affiliations:** Ithaca, N. Y.


### ADDRESS AT THE INAUGURATION OF THE NEW YORK STATE VETERINARY COLLEGE, SEPTEMBER 24, 1896.

By PROFESSOR JAMES LAW,
ITHACA, N. Y.

It seems desirable to say a few words to you collectively, in view of the inauguration of a new enterprise in America—a State Veterinary College. As an English-speaking people, we have been especially influenced by English example in shaping many of our institutions, and in none more so than in those to which veterinary education has been committed. It has been a crowning glory of the Anglo-Saxon races that they have suspected and frowned upon a too paternal government. In Europe and America, in South Africa, Australia, and New Zealand, a prominent aim has been to restrict the functions of government to the protection of the citizen in his personal rights of property and conscience, in his lawful business enterprises, and his pursuit of pleasure. Education, it is true, came in for a constantly increasing share of national control and support, but this was for long mainly along classic lines, and was a legacy that came down to us from the early monastic and ecclesiastical schools. For purely secular education money was slowly and grudgingly allowed, with a wholesome dread of the evils to be apprehended from class legislation. That instinct of even-handed justice which demanded for the citizen a trial by a jury of his peers naturally recoiled from any proposition which looked like an appropriation of public money for the creation or benefit of any special class or guild. It is only in recent years that the manifest value to the nation in its competition with other nations of the highest knowledge and skill in science and arts has led to the founding and support of technical and professional schools of all kinds, to keep the country in the forefront of the race of civilization and progress. As the Anglo-Saxon peoples have gradually awakened to the need of government provision for technical education, those branches which seemed to be of the greatest material value were naturally the first and most liberally dealt with, while those in which the prizes were smaller or the triumphs less striking and competition less close were still left to shift for themselves.

In Great Britain there has never been a State Veterinary College, and the four existing schools have been all founded by private enterprise and conducted independently of State grants. In America, as in England, the veterinary schools have been private ventures, and consequently largely dominated by the financial results. The founders of such schools were met at the threshold by the imperative questions: "Will the venture pay? Can we secure fees enough to sustain it? Will the name of the college bring us greater and more remunerative practice? Will the prospective fees, fame, and practice warrant the investment?"

The answer is necessarily dominated by the question of money, and the temptation is great to subordinate the educational consideration. The pressure is heavy: 1. To shorten the curriculum; 2. To admit ill-prepared candidates; 3. To graduate large numbers irrespective of fitness; 4. To further abridge the already short course; and as a final degradation, 5. To sell diplomas. To this last, lowest depth of sordidness more than one veterinary college in America has sunk. But short of this, even the surviving and honorable colleges have been one and all prevented from achieving the status which the nature of the subject demanded. The preliminary education and the trained mind, which are requisite to the pursuit of science, have not been required for matriculation, and the course has been abridged to such an extent that even a trained mind cannot successfully cover the required ground in the time allowed. Meanwhile the field of veterinary science has been rapidly enlarging, deepening, widening, and becoming more thorougly cultivated, so that the insufficiency of the untrained student and short curriculum has become more and more marked year by year.

The contrast with the schools of veterinary medicine on the continent of Europe will emphasize this statement. In entering a Continental veterinary school the student must show that he has graduated from a *real skule*, *gymnasium*, or *college*, and he must pursue a veterinary course of from three years and a half to five years of nine or ten months each ere he can hope to secure a degree. Add to this that the great advances in medicine have been such that the majority of the students have to study an additional year ere they can secure the coveted diploma, and we can appreciate the hopeless inadequacy of a course of two or even of three sessions of five or six months each, which has not been preceded by a mental training in high school or college.

These continental veterinary colleges would have been not more thorough than the English or American had they been dependent on private enterprise. But there is no veterinary college on the continent of Europe to-day that is not a ward of the government. Each one has been founded and is sustained by the commonwealth, just as are the army, the navy, the experiment farm, etc.

This paternalism is founded on a long experience of their value, of which I may be permitted to give a single example. The disease rinderpest, which confines its ravages to ruminants, and, as its name indicates, almost entirely to cattle, formerly spread over most of Europe at frequent intervals, killing 20 to 95 per cent. of the bovine race at a single invasion. Paulet tells us that in Western Europe in three years (1711–14) it cut off 1,500,000 head of cattle, and Faust says that in the whole of Europe in four general invasions, dating from 1711, it destroyed not less than 200,000,000 head. At $20 per head this reaches the astounding sum of $4,000,000,000. So late as 1884, according to Reynal, it destroyed 1,000,000 head in Southern Russia alone. Thanks to the veterinary profession of Europe this disease can never attain such boundless sway, and though still extended at intervals in the course of belligerent armies or in the channels of trade, it is always met with intelligent measures of control and speedily suppressed.

This is but one of the deadly plagues of the Old World, the ruinous extension of which led in 1762 to the establishing of the first European veterinary school at Lyons, France, under the presidency of Bourgelat. This was followed a year later by a second school at Charenton, near Paris, and still later by a third at Toulouse. These were succeeded by a score of others in the different countries of the continent, and all at the national charge and under government control. They are justly looked upon as economic investments, not only for the restriction and extinction of the plague, but also for the conserving of the lives and efficiency of the horses of the cavalry and artillery, for the protection and fostering of the various animal industries, and indirectly, though no less certainly, for the permanent preservation of the fertility of the soil.

The results have abundantly vindicated the wisdom of the investment. The protected herds have furnished a cheap and abundant food for the growing population. The increasing demand for cattle food and the multiplying of the natural sources of rich manure have combined to enrich the fields and improve the crops. The improved agriculture and abundance of food-products have fostered every branch of manufacture and trade and contributed to a substantial prosperity.

The contrast in countries where veterinary science has been ignored is quite instructive. In South Africa, apart from the mining interests, grazing has long been the main source of wealth. Into this country lung-plague was imported in infected Dutch cattle in 1854, and extending on the unfenced grazing tract, under a semi-torrid climate, proved so disastrous that, according to Lindley, whole herds of one or two hundred head would perish without a single exception. At that time the Matabele chief, occupying land protected on two sides by inaccessible cliffs, successfully defended his passes against the diseased catttle and saved the wealth of his people.

Recently a cargo of cattle from infected Hindostan has implanted the still more redoubtable rinderpest in South Africa, and in the disturbed condition of the country this has penetrated even into Matabeleland, and bids fair to destroy the cattle industry of South Africa.

Again, the lung-plague imported into Australia in 1859, in a diseased English cow, was allowed to spread over the whole island continent, and permanently blighted the cattle industry in one of the finest pasture-lands on earth. In England this same lung-plague in the forty years succeeding 1842 cost the nation $500,000,000. In the United States the same plague prevailed in our eastern seaboard States for over forty years, causing losses that have never been estimated, and incidentally leading to an embargo on American cattle in England which entailed a loss of $10 a head on an average to the exporter. This alone amounted to $2,000,000 per annum. It was only when the plague reached the centre of our cattle traffic (Chicago) and bade fair to invade the whole country, including the unfenced territories, and to repeat in America the experience of South Africa and Australia, that the National and State governments were roused from their lethargy, and we were empowered to take efficient measures for its extinction. Happily now it has no place on this continent, and, with reasonable precaution, can never make a new invasion. The same line of thought and similar historic facts could be followed and adduced as to the other animal plagues, including affections caused by the larger animal parasites, as to enzoötic diseases caused by faulty conditions of the environment, as to constitutional diseases due to errors in breeding, diet, and regimen, and as to local diseases, many of which are due to improper treatment.

In America, as in Europe, we can successfully maintain that the benefits already drawn from the veterinary profession have abundantly vindicated its claim to State support. But the prospective value of the work of veterinary investigation and education far exceeds all that it may have accomplished for the nation in the past. Among our horses glanders yearly claims a large and valuable contribution to its devouring poison. Among cattle, anthrax, tuberculosis, and southern cattle fever cause widespread though needless destruction. Among sheep, flocks are decimated everywhere by remorseless parasites, internal and external. Among swine, the preventable infectious fevers cost the nation, on a low estimate, 20,000,000 per annum. Among fowls, the prevalent contagious affections are no less disastrous.

In the matter of numbers, the wealth at stake in the live-stock of America is as great as that of European nations, and, to the reasoning mind, is not less worthy of measures for its protection. In four of the most important countries of Western Europe the aggregate of the farm mammals is considerably less than that of the United States. Yet these four countries of Western Europe (France, Belgium, Holland, and Germany) have eleven veterinary schools maintained and fostered at State expense. Surely our own Empire State, with its 9,500,000 of farm mammals, with its large emporia at Buffalo, Albany, and New York for the reception and diffusion of live-stock from other States, and its record of a recent riddance from a cattle plague which for over forty years had hung like a pall on the cattle industry of the State, and exacted a tax of two millions or more per annum from home herds and exports, is fully justified in establishing a State Veterinary College.

(To be continued.)

